# Targeting lung cancer through inhibition of checkpoint kinases

**DOI:** 10.3389/fgene.2015.00070

**Published:** 2015-02-27

**Authors:** Randi G. Syljuåsen, Grete Hasvold, Sissel Hauge, Åslaug Helland

**Affiliations:** ^1^Department of Radiation Biology, Institute for Cancer Research, Norwegian Radium Hospital, Oslo University Hospital, Oslo, Norway; ^2^Department of Genetics, Institute for Cancer Research, Norwegian Radium Hospital, Oslo University Hospital, Oslo, Norway; ^3^Department of Oncology, Norwegian Radium Hospital, Oslo University Hospital, Oslo, Norway

**Keywords:** checkpoint abrogation, lung cancer, ATR, Chk1, Wee1, replication stress, cancer stem cells, hypoxia

## Abstract

Inhibitors of checkpoint kinases ATR, Chk1, and Wee1 are currently being tested in preclinical and clinical trials. Here, we review the basic principles behind the use of such inhibitors as anticancer agents, and particularly discuss their potential for treatment of lung cancer. As lung cancer is one of the most deadly cancers, new treatment strategies are highly needed. We discuss how checkpoint kinase inhibition in principle can lead to selective killing of lung cancer cells while sparing the surrounding normal tissues. Several features of lung cancer may potentially be exploited for targeting through inhibition of checkpoint kinases, including mutated p53, low ERCC1 levels, amplified Myc, tumor hypoxia and presence of lung cancer stem cells. Synergistic effects have also been reported between inhibitors of ATR/Chk1/Wee1 and conventional lung cancer treatments, such as gemcitabine, cisplatin, or radiation. Altogether, inhibitors of ATR, Chk1, and Wee1 are emerging as new cancer treatment agents, likely to be useful in lung cancer treatment. However, as lung tumors are very diverse, the inhibitors are unlikely to be effective in all patients, and more work is needed to determine how such inhibitors can be utilized in the most optimal ways.

## INTRODUCTION

Lung cancer is difficult to treat. Its frequent incidence combined with the low success rate of current treatment strategies, make lung cancer the overall deadliest form of cancer worldwide (Siegel et al., 2012). Although recent progress has demonstrated druggable driver mutations in lung cancer, such as ALK (Anaplastic Lymphoma Kinase) translocations and EGFR (Epidermal Growth Factor Receptor) mutations, these are found only in a small subset of all lung cancer patients, and treatment resistance develops invariable (Chen et al., 2014). Most patients are diagnosed in late stages of the disease and are treated with chemotherapy or radiotherapy, with symptomatic and sometimes life prolonging effect. Overall, 5 years survival is bleak, approaching 18% (National Institutes of Health, 2011; Cancer Registry of Norway, 2012). There is therefore still a strong need for development of new treatment strategies in lung cancer.

In response to DNA damage or replication stress, activation of the checkpoint kinases Chk1 (Checkpoint kinase 1), Wee1 and ATR (Ataxia Telangiectasia and Rad3 related) facilitate S and G2 checkpoint arrest (Sanchez et al., 1997; Liu et al., 2000; Wang et al., 2001; Heffernan et al., 2002; Sørensen et al., 2003; Beck et al., 2012). These kinases may promote survival of tumor cells both in the absence and presence of DNA damaging agents. Inhibitors of these kinases have been developed and are currently in pre-clinical and clinical testing for cancer treatment (Do et al., 2013; Llona-Minguez et al., 2014; McNeely et al., 2014). For instance, several clinical trials are ongoing with the Wee1 inhibitor MK1775 (AZD1775) for combined treatment with radiation therapy or chemotherapy. These studies are performed in several cancer types, including lung cancer. Trials are also ongoing with the Chk1-inhibitors LY2606368 and SCH 900776 as single agents or in combination with chemotherapeutic drugs (ClinicalTrials.Gov). Of note, non-small cell lung cancer (NSCLC) patients treated with Chk1 inhibitors reportedly showed partial responses in Phase 1 trials (Calvo et al., 2014; Sausville et al., 2014). The first clinical trials with ATR inhibitors were recently initiated, evaluating the safety and biological effects of AZD6738 and VX-970 (ClinicalTrials.Gov).

Here, we briefly review the rationales for using checkpoint kinase inhibitors as anticancer agents, and discuss their potential for treatment of lung cancer. The focus is on how checkpoint kinase inhibition in principle can lead to selective killing of lung cancer cells while sparing the surrounding normal tissue.

## GENERAL PRINCIPLES BEHIND THE TUMOR SELECTIVE EFFECTS OF Chk1/ATR/Wee1 INHIBITORS

### G2 CHECKPOINT ABROGATION

Following DNA damage, the G2 checkpoint prevents mitotic entry of damaged cells and thereby protects against mitotic catastrophe and cell death (Syljuåsen et al., 2004). The G2 checkpoint is activated mainly through inhibition of the mitosis promoting complex Cyclin B-Cdk1 (Cyclin dependent kinase 1). Wee1 kinase directly phosphorylates Cdk1 on its Tyrosine 15 residue, an inhibitory phosphorylation site negatively regulating Cdk1 activity (Parker and Piwnica-Worms, 1992). Tyrosine 15 phosphorylation is counteracted by the CDC25 (Cell Division Cycle 25) phosphatases, which in turn are negatively regulated by Chk1 (Sanchez et al., 1997). The activity of Chk1 is stimulated by ATR-mediated phosphorylation of Chk1 at the Serine 317 and 345 residues (Zhao and Piwnica-Worms, 2001). Thus, inhibition either of Wee1, Chk1, or ATR leads to decreased inhibitory phosphorylation of Cdk1 and thereby increased Cdk1 activity and G2 checkpoint abrogation.

**FIGURE 1:**
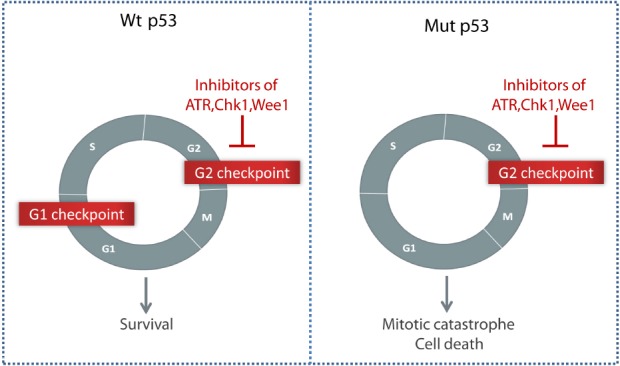
**Selective killing of p53 negative tumor cells through G2 checkpoint abrogation by inhibitors of ATR, Chk1, or Wee1.** Compared to normal cells with an intact G1 checkpoint, cancer cells lacking the p53-dependent G1 checkpoint may depend more on the G2 checkpoint to survive after DNA damage.

Importantly, it was hypothesized that cancer cells lacking the G1 checkpoint may depend more on the G2 checkpoint for cell survival (reviewed in Dixon and Norbury, 2002; Ma et al., 2011). The G1 checkpoint is activated through the function of the tumor suppressor p53, and is often absent in cancer cells due to p53 mutations or other defects in the p53 signaling pathway (Nagasawa et al., 1995). Abrogation of the G2 checkpoint by inhibitors of Chk1, Wee1, or ATR may therefore selectively sensitize p53 defective cancer cells to DNA damaging agents, while the surrounding normal cells could be spared (Figure 1; Leijen et al., 2010; Ma et al., 2011; Hirokawa et al., 2014).

### S PHASE DAMAGE

While Chk1, Wee1, and ATR are widely known as key regulators of the G2 checkpoint, these kinases also regulate CDK activity during S phase, and thereby prevent the induction of DNA damage during normal S phase progression (Syljuåsen et al., 2005; Sørensen and Syljuåsen, 2012). Increased CDK activity in response to checkpoint kinase inhibition promotes unscheduled replication initiation, leading to nucleotide shortage, replication stalling and subsequent activation of endonucleases and DNA breakage (Beck et al., 2012). In addition, shortage of other replication factors such as RPA (Replication Protein A) contributes to replication fork collapse after the unscheduled initiation (Toledo et al., 2013). ATR and Chk1 also play a more direct role in stabilizing stalled replication forks, by mechanisms that are still poorly understood (Brown and Baltimore, 2003; Friedel et al., 2009), but may involve suppression of nucleases (Froget et al., 2008; Forment et al., 2011). Thus, Chk1, ATR, and Wee1 inhibitors do not only cause G2 checkpoint abrogation, but also induce DNA damage in S phase, which may contribute to the cytotoxic effects of these inhibitors (Toledo et al., 2011; Sørensen and Syljuåsen, 2012).

**FIGURE 2:**
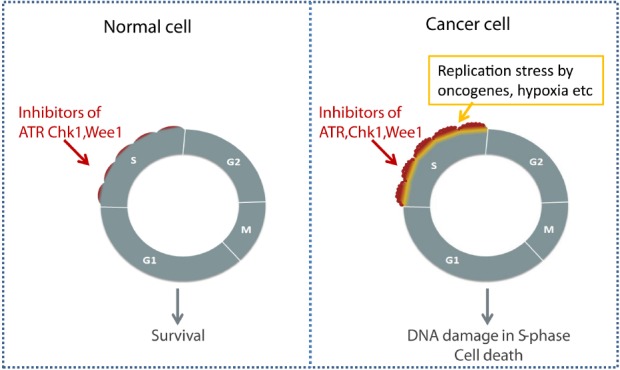
**Selective killing of cancer cells through S phase damage induced by checkpoint kinase inhibitors.** Elevated replication stress in cancer cells due to oncogenes or hypoxia may lead to increased cytotoxic effects of checkpoint kinase inhibitors in S phase.

During tumor development, the expression of oncogenes, such as Cyclin E, Myc and Ras, may abnormally increase replication, leading to so-called “replication stress” (Bartkova et al., 2005; Gorgoulis et al., 2005; Halazonetis et al., 2008). Importantly, cancer cells with elevated replication stress activate ATR/Chk1 and may depend more on these kinases for cell survival compared to normal cells (Gilad et al., 2010). In such cells ATR/Chk1 may help restrain the CDK activity and replication to tolerable levels, and these cells also likely depend more on Wee1. When combined with ongoing replication stress caused by oncogenes, checkpoint kinase inhibitors may therefore cause cytotoxic levels of S phase damage in tumor cells, while having minimal effects on normal cells. In addition to G2 checkpoint abrogation in p53 defective cells, increased S phase damage thus represents another reason for tumor-selective effects of Chk1, ATR, and Wee1 inhibitors (Figure 2; Sørensen and Syljuåsen, 2012; Do et al., 2013; Lecona and Fernandez-Capetillo, 2014).

### INHIBITION OF HOMOLOGOUS RECOMBINATION REPAIR

Another shared function of Chk1, Wee1, and ATR is their role in positive regulation of homologous recombination (HR) repair, a major pathway for repair of DNA double strand breaks. In fact, inhibition of HR repair was suggested as a main mechanism for the radiosensitizing effects of the Chk1-inhibitor AZD7762, besides G2 checkpoint abrogation (Morgan et al., 2010). The Chk1-mediated regulation of HR repair occurs at least partly through direct phosphorylation of the Rad51 recombinase repair protein (Sørensen et al., 2005). A recent study showed that Wee1 inhibition also can inhibit Rad51 function and HR repair (Krajewska et al., 2013). The increased CDK activity after Wee1 inhibition leads to phosphorylation of BRCA2 at the 3291 residue, which in turn inhibits Rad51 loading (Davies and Pellegrini, 2007; Krajewska et al., 2013). The role of ATR in HR repair is less clear. However, ATR may support HR repair through control of the S phase checkpoint allowing time for repair, and through phosphorylation of Chk1 or other factors such as BRCA1 (Brown et al., 2014).

Notably, HR repair is largely restricted to S and G2 phase cells (Jeggo et al., 2011), and inhibition of HR repair will thus not affect non-cycling G0 or G1 phase cells. As tumors typically contain more cycling cells compared to the surrounding normal tissues, inhibition of HR repair in S and G2 phase cells therefore likely contributes to promote tumor selective effects of checkpoint kinase inhibitors.

### CANCER-ASSOCIATED CHANGES IN ATR, Chk1, OR Wee1 EXPRESSION

ATR, Chk1, and Wee1 are all essential proteins required for embryonic development in mice (Brown and Baltimore, 2000; Liu et al., 2000; Tominaga et al., 2006). Consistent with an essential role, homozygous inactivating mutations of the genes encoding these checkpoint kinases have not been observed in cancer. However, a small subset of human tumors shows heterozygous mutations in ATR or Chk1 (Bertoni et al., 1999; Lewis et al., 2005; Zighelboim et al., 2009), resulting in reduced protein expression. To our knowledge mutations in Wee1 have not been reported. However, Wee1 may be downregulated through other mechanisms such as cancer-associated expression of microRNAs (Butz et al., 2010; Tili et al., 2011). Interestingly, a recent siRNA screen identified ATR itself, and regulators of ATR kinase activity, among the factors protecting cells against the ATR inhibitor VE821 (Mohni et al., 2014). Cancer cells with reduced expression of ATR were thus more sensitive to the ATR inhibitor. This is likely because of more complete ATR inactivation in response to concentrations of VE821 that normally would be sufficient to only partially inactivate the cellular pool of ATR. Hence, it is possible that cancer cells with inherent reduced expression of ATR, Chk1, or Wee1 may respond to low concentrations of checkpoint kinase inhibitors, whereby normal cells could be spared.

On the other hand, ATR, Chk1, and Wee1 are also overexpressed in a subset of human cancers (Iorns et al., 2009; Mir et al., 2010; Cole et al., 2011; Magnussen et al., 2012; Parikh et al., 2014). In some cases, the checkpoint kinases may be upregulated as part of a cellular response to cope with elevated replication stress (Sørensen and Syljuåsen, 2012; Lecona and Fernandez-Capetillo, 2014). For instance, Myc amplification has been linked with elevated Chk1 levels and increased sensitivity to Chk1 inhibitors (Cole et al., 2011; Hoglund et al., 2011). Possibly, such cells will therefore depend on the high levels of ATR, Chk1, or Wee1 to survive. Inhibitors of ATR, Chk1, or Wee1 may thus potentially be more toxic to cancer cells inherently expressing high levels of these kinases. Taken together, this creates a complex picture where either abnormal *low* expression, or *high* expression, of ATR, Chk1, or Wee1 in cancer cells may potentially cause increased sensitivity to inhibitors of these checkpoint kinases.

### TUMOR HYPOXIA

Hypoxia is very common in solid tumors and develops due to rapid growth of cancer cells and insufficient growth of new blood vessels, resulting in higher oxygen consumption than supply. Tumors can contain regions of long-term, persistent hypoxia, as well as regions with fluctuations in oxygen leading to cycles of transient hypoxia and reoxygenation (Bertout et al., 2008; Dewhirst, 2009). Hypoxia is a poor prognostic factor and is associated with resistance to conventional cancer therapy (Bristow and Hill, 2008; Horsman et al., 2012; Luoto et al., 2013; Walsh et al., 2014). However, hypoxic tissues also offer the advantage of being distinct from the surrounding normal tissues, and as such may be exploited to obtain selective killing of cancer cells. Importantly, severe hypoxia leads to replication stress and activation of DNA damage checkpoint signaling (Hammond et al., 2002, 2003). Therefore, inhibitors of ATR or Chk1 may in fact represent hypoxic cell cytotoxins (Hammond et al., 2004). Indeed, several studies have demonstrated increased cytotoxic effects of both Chk1 and ATR inhibitors in cancer cells exposed to hypoxia compared to normoxic cells (Hammond et al., 2004; Pires et al., 2012; Cazares-Korner et al., 2013; Hasvold et al., 2013). However, the increased effects of Chk1 inhibitors were observed after reoxygenation following prolonged hypoxic exposure, and not when the Chk1 inhibitors were present only during hypoxia (Hasvold et al., 2013). Chk1-inhibitors may thus be more effective combined with other treatments that cause reoxygenation, such as for instance fractionated radiotherapy. The impact of hypoxia on the effects of Wee1 inhibitors is not clear and largely awaits investigation.

Although more work is needed to elucidate the influence of a hypoxic tumor microenvironment on the responses to checkpoint kinase inhibitors, these studies do indicate that hypoxic tumors may be more sensitive to checkpoint kinase inhibitors compared to the surrounding normoxic tissue.

### CANCER STEM CELLS

Intra-tumor heterogeneity may play an important role during cancer treatment. Particularly, small sub-populations of tumor-initiating cells, or cancer stem cells (CSCs), may survive cancer therapy and promote tumor regrowth. Although the characterizing markers (Keysar and Jimeno, 2010) and origin of these cells has been a matter of debate, their existence in human cancers is now mainly accepted (O’Connor et al., 2014). Due to their inherent resistance against conventional cancer treatments and important role in tumor recurrence and metastasis, finding strategies for eradicating these CSCs is a crucial task.

Interestingly, several studies have demonstrated that DNA damage-induced signaling is enhanced in CSCs of various origins (glioblastoma, NSCLC, head and neck, prostate and pancreas), including increased activation of Chk1, and such cells are particularly sensitive to Chk1-inhibitors (Bao et al., 2006; Bartucci et al., 2012; Venkatesha et al., 2012; Wang et al., 2012; Wu et al., 2012; Fang et al., 2013; Bertrand et al., 2014; Signore et al., 2014). Furthermore, inhibition of ATR has been shown to cause depletion of chemoresistant and tumorigenic CD133^+^ colon cancer cells (Gallmeier et al., 2011), and Wee1 inhibition radio-sensitized glioblastoma stem cells *in vitro* (Mir et al., 2010). The expression of Wee1 was in fact higher in CD133^+^ compared to CD133^–^ primary glioblastoma cells (Mir et al., 2010), and Wee1 was among the most downregulated genes upon differentiation of PTEN positive glioblastoma stem cells (Forte et al., 2013), indicating that high levels of Wee1 may be required to maintain a stemlike state. However, another report found no radio-sensitization by the Wee1 inhibitor MK1775 in glioblastoma neural stem cells (Sarcar et al., 2011).

More work is needed to clarify the effects of Chk1 versus ATR and Wee1 inhibition in CSCs, and to understand the mechanisms involved. Reports regarding the repair capacity of CSCs have been conflicting (Bao et al., 2006; McCord et al., 2009; Ropolo et al., 2009), and the effectiveness of Chk1 inhibition in such cells has primarily been coupled to regulation of cell cycle progression and cell death through apoptosis and mitotic catastrophe (Ropolo et al., 2009).

## CHALLENGES OF LUNG CANCER TREATMENT

Excellent reviews summarizing and discussing the various therapies and targets of lung cancer in depth have been published elsewhere (Willers et al., 2013; Berge and Doebele, 2014; Chen et al., 2014), and we therefore only briefly summarize some of the main challenges of current lung cancer treatment below. These challenges are relevant with respect to evaluating the potential use of checkpoint kinase inhibitors.

Lung cancer is a common disease, and the number one killer among cancers (Brustugun et al., 2014). However, there exists a huge diversity, both in clinical manifestation and patients. While most patients are or have been daily smokers, some have never smoked. Many patients are old, but some patients get this diagnosis at younger age. Traditionally lung cancers were divided in small cell lung cancer and non-small cell lung cancers. Current treatment algorithms require both histological subtype (adenocarcinoma vs. squamous cell carcinomas) and analyses for specific genetic aberrations. Treatment and follow-up of lung cancer patients vary depending on these specific characteristics (Chen et al., 2014).

Approximately 75% of lung cancer patients are diagnosed with stage four disease, and receive palliative treatment with chemotherapy and/or radiotherapy. The standard therapy is a platinum (cisplatinum or carboplatinum) combined with a second drug (gemcitabine, pemetrexed, or vinorelbine for instance). The effects are unfortunately not long lasting, and new strategies are needed for a more effective treatment (Bonanno et al., 2014).

A subset of patients is treated with targeted therapy based on genetic aberrations in the tumor. Approximately 10–15% of NSCLCs are mutated in the EGFR gene, more common in Asian populations and among never-smokers. Patients with an EGFR-mutation in their tumor cells are effectively treated with tyrosine kinase inhibitors like gefitinib, erlotinib, or afatinib. These drugs have effects for 8–9 months in median, and second and third line drugs are in development (Melosky, 2014).

A small percentage of the tumors have a translocation involving the ALK-gene. This is present in approximately 2–6% of the adenocarcinomas, and is also effectively treated with targeted therapy (crizotinib, ceritinib; Chia et al., 2014). Unfortunately, resistance develops in all patients. Other genetic alterations are currently being tested in clinical studies. BRAF mutations, ROS1 translocations, PIK3CA mutations, MET amplifications and HER2 aberrations are examples of such alterations, present in only a small percentage of lung cancers and currently being targeted in clinical studies (Chen et al., 2014).

Unfortunately, while lung cancer treatment today can relieve symptoms and prolong life with some months, the disease usually progresses. More knowledge is therefore needed about mechanisms underlying disease progression in order to develop new treatment strategies.

## THE POTENTIAL OF ATR/Chk1/Wee1 INHIBITORS FOR TREATMENT OF LUNG CANCER

**FIGURE 3:**
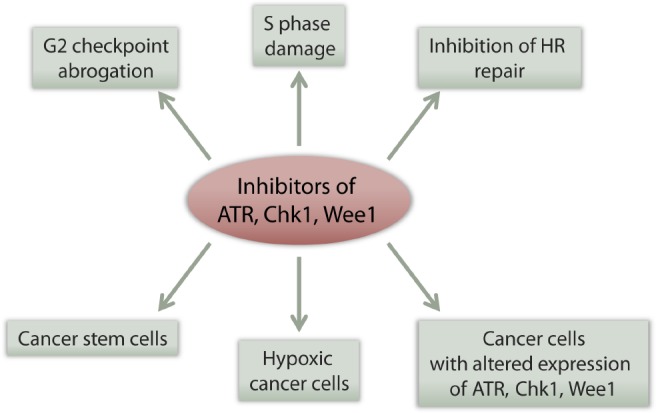
**Multiple effects of checkpoint kinase inhibitors can potentially contribute to their tumor selectivity.** See main text for details.

In light of the general principles behind the tumor selectivity of ATR, Chk1, and Wee1 inhibitors outlined above (summarized in Figure 3), there are several specific traits associated with lung cancer that may potentially increase the efficacy of such inhibitors. Below we outline these traits and discuss relevant published experimental work.

### p53 MUTATIONS

Firstly, p53 mutations are very common in lung cancer (Takahashi et al., 1989). This is important as loss of p53 is proposed as a major reason behind the tumor specific effects of checkpoint kinase inhibitors (see above). Previous work showed that p53 disruption could sensitize p53 wt lung cancer cells (A549 and LXSN) to the combined effects of radiation and the Chk1-inhibitor UCN-01 (Xiao et al., 2002). Similarly, the Wee1 inhibitor MK1775 radio-sensitized lung cancer cells (A549, H460, H1299) in a p53-dependent manner (Bridges et al., 2011). Furthermore, siRNA mediated depletion of p53 sensitized A549 lung cancer cells to the ATR inhibitor VE821 in combination with cisplatin (Reaper et al., 2011), and A549 cells depleted of p53 were also sensitized to another ATR inhibitor, VX-970, in combination with various DNA damaging drugs (Hall et al., 2014). These results thus support the hypothesis that inhibitors of ATR, Chk1, or Wee1 can be used to selectively target p53 deficient lung cancer cells. However, although p53 status has proven important for the effects of checkpoint kinase inhibitors in isogenic cell systems, p53 status alone does not seem sufficient to predict responses across large heterogenic cancer cell panels (Petersen et al., 2010; Guertin et al., 2013; Hall et al., 2014). Particularly, the cytotoxic effects of ATR, Chk1, or Wee1 inhibitors given as single agents vary between different cell lines regardless of p53 status (Petersen et al., 2010; Guertin et al., 2013; Hall et al., 2014). It is therefore unlikely that the p53-status alone can fully predict the efficacy of ATR, Chk1, and Wee1 inhibitors in lung cancer patients. However, p53 deficiency is, one among several factors, contributing to increasing the efficacy of these inhibitors.

### INCREASED REPLICATION STRESS CAUSED BY GENETIC ALTERATIONS OR HYPOXIA

Secondly, replication stress is a common feature of lung cancer, which could sensitize to checkpoint kinase inhibition by enhancing the S phase damage (see above). For instance, the Myc oncogene is an inducer of replication stress, and some lung cancers are Myc-driven (Little et al., 1983). Exogenous overexpression of Myc caused increased sensitivity to Chk1 inhibitors in various cell types (Cole et al., 2011; Hoglund et al., 2011; Murga et al., 2011). In addition, ATR inhibitors caused increased cell death in Myc overexpressing cells, and partial genetic depletion of ATR prevented growth of Myc-induced tumors in mice (Murga et al., 2011; Schoppy et al., 2012). Thus, Myc overexpression may sensitize to both Chk1 and ATR inhibitors. Furthermore, Ras is mutated in a subset of lung cancers (Vasan et al., 2014). Oncogenic Ras can cause replication stress and increase the efficacy of ATR inhibitors (Gilad et al., 2010; Schoppy et al., 2012), and the Wee1 inhibitor MK1775 was identified in a screen for agents targeting Ras driven malignancies (Weisberg et al., 2014).

In addition, a proportion of NSCLCs reportedly show reduced expression of the repair protein ERCC1 (Postel-Vinay et al., 2012; Wei et al., 2012). Low levels of ERCC1 sensitize cells to platinum-based drugs such as cisplatin, and ERCC1 is currently being tested as a predictive biomarker for cisplatin-based chemotherapy in lung cancer (Postel-Vinay et al., 2012; Wei et al., 2012; Bonanno et al., 2014), although the methods of evaluating the ERCC1 levels have been questioned (Friboulet et al., 2013). Interestingly, a recent siRNA screen for factors protecting against the ATR inhibitor VE821 identified ERCC1 among the strongest hits (Mohni et al., 2014). Cells with low levels of ERCC1 ceased S phase progression and showed increased cell death after ATR and Chk1 inhibition (Mohni et al., 2014). Lung cancer cells with low levels of ERCC1 may therefore be highly sensitive to ATR, as well as Chk1, inhibitors.

Thus, manipulation of Myc, Ras or ERCC1 in various cell systems can cause altered sensitivity to ATR, Chk1, and Wee1 inhibitors. However, it remains to be shown whether Myc, ERCC1 and/or Ras status can predict responses to checkpoint kinase inhibitors across large panels of heterogenic human lung tumors. Potentially, these factors could be valuable as predictive biomarkers for responses to checkpoint kinase inhibitors *in vivo*.

Moreover, hypoxia is common in lung tumors (Bollineni et al., 2012). Hypoxia can induce replication stress (Hammond et al., 2003) and may sensitize to ATR or Chk1 inhibitors (Olcina et al., 2010). Nonetheless, few studies have focused on hypoxia and the effects of checkpoint kinase inhibition in lung cancer. Of note, a recent report demonstrated decreased viability of hypoxic A549, H1299, and H1975 lung cancer cell lines after treatment with a hypoxia-activated Chk1 inhibitor (the CH-01 prodrug; Cazares-Korner et al., 2013), indicating that hypoxic lung tumors may be sensitive to Chk1 inhibitors. In contrast, a single study suggested that hypoxia does not sensitize H1299 lung cancer cells to the Wee1 inhibitor MK1775 (O’Brien et al., 2013).

### LUNG CANCER STEM CELLS

Though less studied than CSCs in glioblastoma, several studies have suggested that lung tumors contain sub-populations of such tumor initiating cells (reviewed in Singh and Chellappan, 2014). High expression levels of CSC markers such as CD133 and CD44 have been identified as poor prognostic factors in NSCLC patients (Luo et al., 2014; Wu et al., 2014), and studies with lung cancer cell lines have confirmed the presence of side population (SP) cells and spheroid-forming cells with typical CSC properties, including resistance to chemotherapy agents and radiation (Ho et al., 2007; Salcido et al., 2010; Fang et al., 2013; Lundholm et al., 2013). Furthermore, recent studies have shown that lung cancer cell lines surviving radiation express higher levels of several CSC markers such as CD44 or CD24 (Gomez-Casal et al., 2013).The cell adhesion molecule CD44 in particular was upregulated in cells surviving radiation from two different lung cancer cell lines (Gomez-Casal et al., 2013), suggesting that this marker may be associated with radiation resistance. CD44 positive cells were also found to be resistant to cisplatin in a study of NSCLC cell lines (Leung et al., 2010).

Overcoming such treatment resistance is vital for successful treatment of lung cancer patients, and a few recent studies indicate that Chk1 inhibition might be a promising way to do so. In spheroid-forming cells derived from the NSCLC cell line NCI-H1299, the combination treatment of Chk1 inhibition and gemcitabine enhanced the antiproliferative effect of gemcitabine, though it failed to deplete the CSC population completely (Fang et al., 2013). Even more promising, in a study using cells derived directly from lung cancer patients, activation of Chk1 in response to chemotherapeutic drugs was strongly enhanced in cells grown as spheres (undifferentiated) compared to adherent cells grown in a monolayer (differentiated; Bartucci et al., 2012). These undifferentiated cells, termed NSCLC-SCs, were also resistant to the cytotoxic effects of cisplatin, gemcitabine and paclitaxel, consistent with a strong repair capacity and checkpoint activation. However, inhibition of Chk1 abolished this chemotherapy resistance, and the combination of chemotherapy and Chk1 inhibitors severely decreased the colony-forming ability of these cells, making Chk1 inhibition a promising strategy for the selective targeting of such NSCLC-SCs. The effects of ATR and Wee1 inhibitors in this context are not known.

### ALTERED EXPRESSION LEVELS OF CHECKPOINT KINASES IN LUNG CANCER

Only limited information is available regarding the expression levels of ATR, Chk1, and Wee1 in lung cancer. However, ATR and Chk1 may be amplified in a subset of genomic unstable lung cancers (Krajewska et al., 2014). In one report, lung cancer cell lines expressing high levels of Chk1 were hypersensitive to Chk1 inhibitors, suggesting that their growth depended on the high amount of Chkl (Grabauskiene et al., 2013). To our knowledge, ATR and Chk1 are not commonly mutated in lung tumors (http://cancergenome.broadinstitute.org). However, other mechanisms of inactivation, like methylation or microRNA-regulation, might play a role. Loss of Wee1 has been reported in NSCLC (Yoshida et al., 2004), but it is not known whether these cells show altered sensitivity to Wee1 inhibitors.

### SYNERGY WITH CONVENTIONAL LUNG CANCER TREATMENTS

While checkpoint kinase inhibitors may show antitumor activity as single agents, they will most likely be used in combination with other treatments. As described above, the current standard treatments of lung cancer include several chemotherapeutic drugs and radiation therapy. Some of these conventional treatments may synergize with checkpoint kinase inhibitors. Multiple studies in different cancer types suggest that ATR and Chk1 inhibitors strongly synergize with gemcitabine and cisplatin (Lecona and Fernandez-Capetillo, 2014; McNeely et al., 2014). This has also been shown in lung cancer. Combination of the Chk1 inhibitor AZD7762 with gemcitabine or cisplatin suppressed growth of lung carcinoma xenografts in mice (Bartucci et al., 2012). H1299 lung cancer cells grown as spheres were resistant to gemcitabine, but could be sensitized by Chk1-inhibition (Fang et al., 2013). In addition, the ATR inhibitor VX-970 sensitized lung cancer cell lines and human lung tumor primary xenografts to cisplatin (Hall et al., 2014). Notably, when comparing the effects of combining the inhibitors with cisplatin or gemcitabine, the ATR inhibitor VX-970 was most effective in combination with cisplatin, and the Chk1 inhibitor AZD7762 in combination with gemcitabine (Hall et al., 2014). Potentiation of the effects of H1299 lung cancer cells to gemcitabine has also been reported with the Wee1 inhibitor MK1775 (Hirai et al., 2009). Furthermore, both Chk1 and Wee1 inhibitors were reported to sensitize lung cancer cells to radiation (Bridges et al., 2011; Yang et al., 2011).

## CONCLUDING REMARKS

**FIGURE 4:**
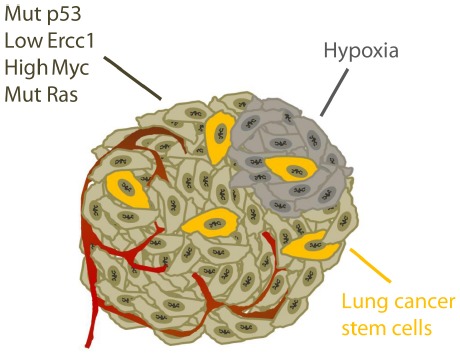
**Specific traits of lung cancer potentially causing tumor selective effects of checkpoint kinase inhibitors.** See main text for details.

In conclusion, lung tumors are difficult to treat and inhibitors of checkpoint kinases ATR, Chk1, and Wee1 will potentially be useful in future treatment strategies. Several common traits of lung cancer can contribute to increase the efficacy of checkpoint kinase inhibitors and promote tumor selective toxicity (summarized in Figure 4). However, as lung tumors are very diverse, the inhibitors are unlikely to be effective in all patients. The main challenges are to identify which patients that would benefit from such treatment and to utilize the inhibitors in the most optimal ways.

The efficacy of checkpoint kinase inhibitors in lung cancer is determined by multiple genetic factors, including p53, Myc, Ras, ERCC1, and the levels of ATR, Chk1, and Wee1 kinases themselves. In addition, the efficacy also depends on other factors, like tumor hypoxia and CSCs. Therefore, it will most likely be difficult to find a single predictive biomarker for responses to checkpoint kinase inhibitors in lung cancer. A combination of several biomarkers may be useful to select patients. In order to identify optimal biomarkers, future studies should aim at understanding mechanisms determining the efficacy of such inhibitors in lung cancer. For instance, the relative contribution of S phase damage versus G2 checkpoint abrogation to the antitumor effects is not well understood. Importantly, the ATR, Chk1, and Wee1 kinases have several distinct functions, which need to be addressed separately. The inhibitors of each of these kinases may therefore be applicable in different situations. Recent preclinical studies have in fact reported synergistic effects when different checkpoint kinase inhibitors were combined, such as for instance Chk1 and Wee1 inhibitors (Carrassa et al., 2012; Russell et al., 2013; Chaudhuri et al., 2014; Chia et al., 2014). The exact mechanism behind this synergy between Chk1 and Wee1 inhibitors is not known, but may likely involve increased S phase damage (Carrassa et al., 2012; Chila et al., 2014). Such combinations should be explored further and be carefully compared to the inhibitors given as single agents at a range of different concentrations.

However, checkpoint kinase inhibitors will most likely be employed in combination with conventional current treatments, such as chemotherapeutic drugs and radiation therapy. Thus, an important issue is how these inhibitors can be utilized in an optimized way together with standard lung cancer treatments. The combined effects of checkpoint kinase inhibitors with chemotherapy and radiation should be further explored in both preclinical as well as clinical lung cancer studies. Particular attention should be given toward potential effects on lung CSCs. As has been shown for other treatment combinations, the sequential treatment timing may also be important (Lund-Andersen et al., 2014). For instance, the optimal time of administrating Chk1 inhibitors in combination with antimetabolites may be after cells have arrested in S phase following the antimetabolite treatment (Grabocka et al., 2014).

Finally, an important issue is whether partial inhibition of checkpoint kinases may increase the risk for the development of genetically unstable normal cells, or potentially lead to more aggressive tumor cells. Few studies have addressed the issue of potential increased genomic instability of cells surviving treatment with checkpoint kinase inhibitors. However, genetic studies from mice suggest that partial, subtle depletion of ATR (by haploinsufficiency) may cause increased genomic instability and accelerate Ras driven carcinogenesis (Gilad et al., 2010). On the other hand, subtle overexpression of Chk1 (by an extra allele of the Chk1 gene) promoted transformation in another report, likely due to increased survival of cells undergoing replication stress (Lopez-Contreras et al., 2012). Low levels of replication stress may therefore allow proliferation of potentially genetic unstable cells, while high levels of replication stress results in cell death. To better evaluate the potential risk associated with checkpoint kinase inhibition, it might be useful to compare the extent of genomic instability in cells surviving after treatment with checkpoint kinase inhibitors with the instability in cells treated with conventional DNA damaging agents.

### Conflict of Interest Statement

The authors declare that the research was conducted in the absence of any commercial or financial relationships that could be construed as a potential conflict of interest.
